# Optimization and Evaluation of Sensor Angles for Precise Assessment of Architectural Traits in Peach Trees

**DOI:** 10.3390/s22124619

**Published:** 2022-06-18

**Authors:** Mugilan Govindasamy Raman, Eduardo Fermino Carlos, Sindhuja Sankaran

**Affiliations:** 1 Department of Biological System Engineering, Washington State University, Pullman, WA 99164, USA; m.govindasamyraman@wsu.edu (M.G.R.); eduardo.carlos@wsu.edu (E.F.C.); 2 Laboratory of Biotechnology, AMG, IDR-IAPAR-EMATER-Agronomic Institute of Paraná, Londrina-PR 86001-970, Brazil

**Keywords:** unmanned aerial vehicle, 3D point cloud, tree height, canopy crown volume, nadir, oblique images

## Abstract

Fruit industries play a significant role in many aspects of global food security. They provide recognized vitamins, antioxidants, and other nutritional supplements packed in fresh fruits and other processed commodities such as juices, jams, pies, and other products. However, many fruit crops including peaches (*Prunus persica* (L.) Batsch) are perennial trees requiring dedicated orchard management. The architectural and morphological traits of peach trees, notably tree height, canopy area, and canopy crown volume, help to determine yield potential and precise orchard management. Thus, the use of unmanned aerial vehicles (UAVs) coupled with RGB sensors can play an important role in the high-throughput acquisition of data for evaluating architectural traits. One of the main factors that define data quality are sensor imaging angles, which are important for extracting architectural characteristics from the trees. In this study, the goal was to optimize the sensor imaging angles to extract the precise architectural trait information by evaluating the integration of nadir and oblique images. A UAV integrated with an RGB imaging sensor at three different angles (90°, 65°, and 45°) and a 3D light detection and ranging (LiDAR) system was used to acquire images of peach trees located at the Washington State University’s Tukey Horticultural Orchard, Pullman, WA, USA. A total of four approaches, comprising the use of 2D data (from UAV) and 3D point cloud (from UAV and LiDAR), were utilized to segment and measure the individual tree height and canopy crown volume. Overall, the features extracted from the images acquired at 45° and integrated nadir and oblique images showed a strong correlation with the ground reference tree height data, while the latter was highly correlated with canopy crown volume. Thus, selection of the sensor angle during UAV flight is critical for improving the accuracy of extracting architectural traits and may be useful for further precision orchard management.

## 1. Introduction

The challenges of a global growing population, simultaneously dealing with unresolved rural poverty in developing areas and the need for better worldwide resource management, have encouraged public awareness in search for healthier and more sustainable practices in crop production. Among them, the methods for achieving higher productivity have been a constant issue in food security forums. Although yield is a result of intrinsic genetics and all conditions given to a plant during the cultivation cycle, precise monitoring of those conditions may lead to better management and higher yields.

For fruit crops, management is also critical, in many cases requiring additional attention for perennial trees. Incorrect management may add years in corrective practices, and thus accurate ongoing monitoring is very important. Fruit crops, including peach trees (*Prunus persica* (L.) Batsch), can be cultivated under different agricultural conditions. In any tree, the canopy is the part exposed to sunlight, and external conditions affect the overall photosynthesis and respiration levels. Thus, it is important to access tree canopy conditions at the right time for better decision-making in orchard management, as well as further actions such as fertilization, spraying, irrigation, pruning, and others [1,2,3]. The architectural features of an orchard include the tree height, canopy area, and crown volume. They provide vital information about tree conditions and help to design precise inputs for fruit production [4].

Traditional orchard practices have provided low degree of information about tree canopies and have led to various problems, including excessive use of chemicals and water [5,6,7] and imprecise overall management. In addition, conventional measurement methods of architectural tree canopy parameters such as height, diameter, area, and volume of crown are laborious and expensive when a high accuracy is required over a larger area [4]. In the present scenario, modern orchard management will be enabled by precise information, which will provide better opportunities to growers to make decisions based on actual data. Thus, there is an urgent need to enhance sensing tools to measure crucial architectural canopy information to strengthen modern and precise orchard management [2].

Remote sensing is widely employed in applications of precision agriculture in orchard management [8]. The main strength of these techniques is to obtain information on large areas at different levels of precision in which different goals can be achieved [9]. Remote sensing platforms such as satellite systems, high and low altitude aircrafts, and unmanned aerial vehicles (UAVs) provide features with different spatial–temporal resolution, surface coverage, and costs. The space-borne satellite system is a promising tool and is generally focused on the long-term monitoring and surveillance of larger areas [10]. Although potentially useful, satellite observations have limited spatial and temporal resolutions and may give a heterogeneous structure of canopy features, leading to difficult evaluations [11]. High and low altitude aircrafts provide a better resolution and deeper level of detail, but usually come with higher preparation efforts regarding flight planning and operational costs [12]. UAVs are small platforms with a low operational cost and higher spatial and temporal resolutions, and are capable of monitoring site-specific areas [13]. Comparing different platforms, a large-area orchard for canopy measurement can be fulfilled through the joint use of UAVs and visual imaging technology [14]. This measurement provides scientific and reliable information for decision-making and brings greater significance to the development of precision orchards [2].

UAVs along with imaging systems can provide data for the digital reconstruction of individual trees in an orchard by computing 3D point clouds, large area orthomosaics, and digital surface and terrain models, providing a basis for orchard canopy measurement. A significant number of studies have been conducted using UAV-integrated sensing systems for precision agriculture and smart farming applications, such as for estimation of the leaf area density and chlorophyll content [15,16,17,18,19,20,21]. Most UAV observations are usually carried out at nadir, where the sensor/camera axis capturing the images is along the vertical direction. The type of image acquisition at nadir is suitable for 2D mapping, but it may not be suitable for 3D mapping and modeling [22]. Oblique images are utilized in the reconstruction of 3D models and achieve better accuracy in terms of both the number of point clouds and measurements [23].

The tree row volume (TRV), total leaf area, and canopy cover were estimated for grapevine and apple orchards using the aerial photogrammetry method with different combinations of sensor angles and flight altitudes (15–60 m flight altitude, 65 and 75° angles) [24]. The measured TRV showed strong a relationship between aerial and ground measurements at the lowest ground sampling distance (GSD) for the grapevines (R^2^ = 0.77 at 0.45 cm pixel^−1^) and apple canopies (R^2^ = 0.82 at 0.90 cm pixel^−1^), whereas increasing the GSD resulted in a weaker relationship for both canopies. Overall, the aerial flight mission with a double grid mission and sensor inclination angle of 65° provided accurate measurements and had greater significance in the development of site-specific higher quality and precise canopy vigor maps [24]. A ground-based 2D and UAV-based 3D model was also used to evaluate the critical architectural traits of apple trees [25]. The extracted traits, such as D_B_S (box-counting fractal dimension), middle branch angle, number of branches, trunk basal diameter, and tree volume, were compared with the ground reference datasets associated with the three training systems (spindle, V-trellis, and bi-axis), two rootstocks, and two pruning methods in the apple trees. The results indicated that D_B_S showed significantly higher values (associated with tree architecture complexity) for spindles training system than the V-trellis training system, and also showed a correlation between the ground and TRV features (total fruit per unit area and trunk area) [25].

In another study, apple orchard canopy parameters such as height and volume based on voxel grid and convex hull techniques were estimated using 3D light detection and ranging (LiDAR) [26]. The 3D point cloud estimated height and volume for apple trees showed a strong correlation with the manually measured height (*r* = 0.84) and volume (*r* = 0.81) [26]. Many studies such as ones described above [24] have collected UAS-RGB imagery at either nadir or oblique angles not lower than 65° and at altitude more than 30 m to measure the tree architectural traits. Most of the research focused on finding the best parameters for flight mission to capture, extract, and achieve the maximum accuracy of the ground measurements. Apparently, no study has yet explored the integration of data collected at different angles combined with low altitude, especially for evaluating the tree architectural traits. However, the reconstruction of high-resolution topography of quarries was performed by combining the nadir and off-nadir imagery, and better accuracies were achieved in the 3D reconstruction of surfaces [27].

Therefore, the overall goal of this study was to digitally reconstruct the architectural traits in peach trees for precise orchard assessment through the optimization and integration of sensor angles using an UAV platform integrated with RGB imagery and a LiDAR ground-based sensing approach.

## 2. Materials and Materials

### 2.1. Study Area and Ground Reference Data

The study was carried out at the Washington State University’s Tukey Horticultural Orchard (46°43′52.88″ N, 117°8′29.09″ W), Pullman, WA, USA. Two rows of peach trees (*Prunus persica* (L.) Batsch) were selected for this study, from which 20 trees were analyzed. Data collection was performed with a UAV integrated with both an RGB camera and a LiDAR system. Similarly, the ground reference data corresponding to the individual tree height, as well as the longitudinal and transversal width data, were manually collected using a measurement scale.

### 2.2. UAV Imagery and LiDAR Data Acquisition

**Figure 1:**
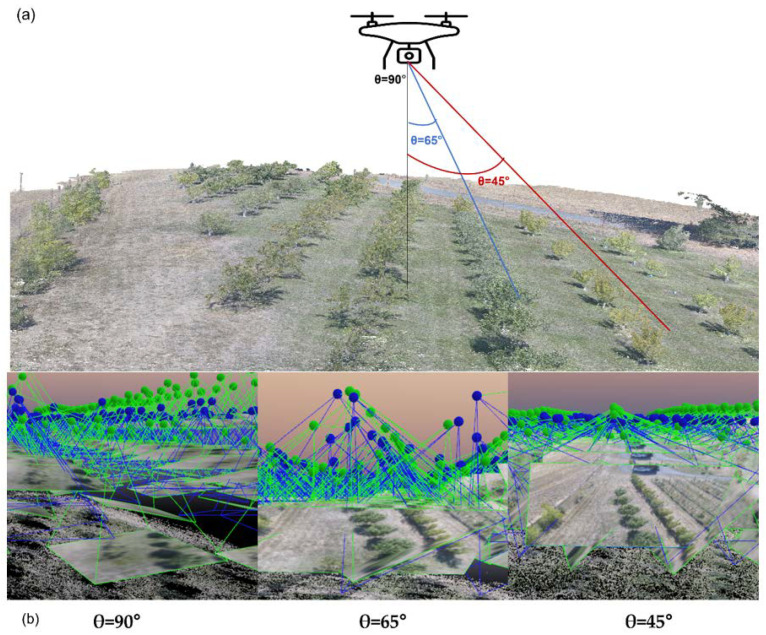
(**a**) Graphical representation of UAV image acquisition for each flight mission at each sensor angle (nadir and oblique angles) and (**b**) different tile points for three different imaging angles. The blue points indicate the GPS positions from the UAV (initial camera position) and the green points are the calibrated positions extracted using the Pix4Dmapper.

The photogrammetry analysis of the UAV data was used for extracting the architectural traits of the peach trees in the orchard. The UAV system (Model: Phantom 4 Pro, SZ DJI Technology Co. Ltd., Shenzhen, China) equipped with RGB camera (SZ DJI Technology Co. Ltd., Shenzhen, China; 20 Megapixel, 84° field of view) was operated using the Pix4Dcapture ground control station. Pix4Dcapture enables planning the flight missions and flight parameters for UAV systems. A total of three missions were set up based on the parameters using the Pix4Dcapture ground control station, including the boundary of the study area, flight path planning, altitude of the flight, speed, and forward and side overlap conditions (Figure 1). All of the three missions were carried out under no cloud conditions. The parameters were the same for all of the three missions except the angle of the sensor inclination (Table 1).

A 3D LiDAR (VLP 16, Velodyne LiDAR, San Jose, CA, USA) system was also used to generate the 3D point cloud of individual trees at ground level. The range of the sensor is 100 m, which produces up to ~600,000 points/s and can acquire a 360° horizontal field view at a distance of 1 m away from a tree, and it has a 30° vertical field of view [26]. The acquired 3D point cloud was saved as .pcap format (Packet Capture) and was visualized using the dedicated software, “Veloview”.

### 2.3. Preprocessing of UAV Images and 3D LiDAR Data

After the image acquisition, the stereo-paired images were stitched using a Pix4Dmapper (Pix4D Mapper, version 4.3.31, Pix4D, Laussane, Switzerland) to generate the 3D point clouds, digital surface model (DSM), digital terrain model (DTM), and orthomosaic images for each individual flight mission. Each mission was processed separately and processed in combination with all of the stereo-paired images acquired from three missions. The pre-processing of UAV datasets using Pix4Dmapper includes computing key points, computing match points, calibration, and matching and point-cloud densification based on the Geotagged information (longitude, latitude, and altitude) of the waypoints. The 3D point cloud (.las format), DSM, DTM, and orthomosaic images for each individual angle and one for the integrated angles (with GSD of 0.40 cm/pixel) were generated for extraction of the architectural features of the individual trees. The integrated dataset can be created by setting the input images as the images acquired at different angles in the Pix4Dmapper software. The .las format 3D point cloud was converted to .csv format using Cloud Compare software to calculate the canopy information. The 3D LiDAR point cloud visualization software, Veloview, which has the capability of analyzing and measuring the 3D point clouds, were used to visualize the live 3D point cloud scenes, allowing the user to select the frame and export it into .csv format.

### 2.4. Extraction of Architectural Features

**Figure 2:**
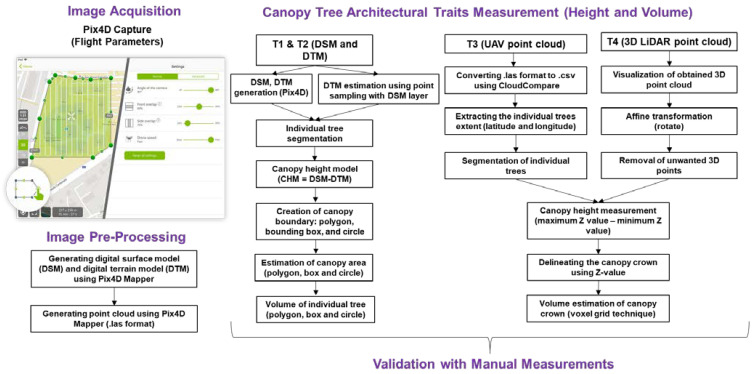
The methodology utilized in this study to extract the canopy architectural traits from the individual trees using UAV and LiDAR data.

This study focused on measuring tree height and canopy crown volume using 2D images and 3D point cloud. The 2D canopy height model utilizing DTM from Pix4D (directly acquired from the software (technique 1, referred to as T1) and DTM (technique 2, referred to as T2) using point sampling from DSM (similar to that reported in [28]) for the individual sensor angle and integrated angle images were generated and used for measuring the tree height and canopy crown volume (Figure 2). In addition, UAV-based point cloud data and LiDAR point cloud data were also used to extract similar features.

#### 2.4.1. Height and Volume Estimation from 2D Datasets

A geospatial processing platform, ArcMap 10.7 (Environmental Systems Research Institute, Redlands, CA, USA), was used to estimate the canopy height model (CHM) by subtracting the digital terrain model (DTM) from the digital surface model (DSM). DSM uses the Earth’s surface including all objects (tree, weeds, trellis, etc.) on it, whereas DTM uses the Earth’s surface without any objects (soil surface as a baseline). In this study, two techniques were used to generate the canopy height model—one was DTM generated from Pix4D (T1 approach) and the other was generated by the point sampling tool using ArcMap 10.7 (T2 approach).

**Figure 3:**
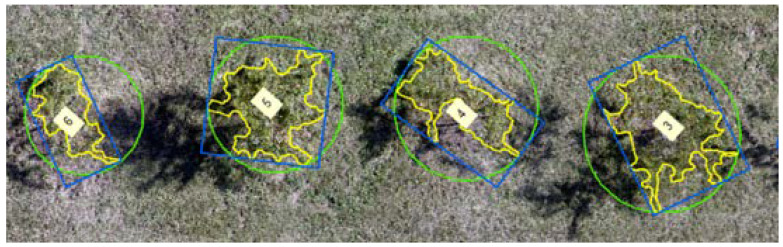
Individual tree boundary (polygon, bounding box, and circle) used to extract the tree height and canopy crown volume.

Using ArcMap 10.7, DTM using the point sampling method was created by plotting random points at the ground surface and extracting the terrain data associated with the points. The extracted point data were interpolated (inverse distance weighing method) to generate a DTM raster. The CHM from both techniques was then reclassified to extract the tree from the other features. The extracted tree canopy area was defined into three shapes, namely polygon, bounding box, and circle (Figure 3). The tree height data were extracted as the maximum height from CHM within the individual tree canopy area within the defined shape using zonal statistics. Similarly, the volume (*V*) of the tree canopy crown was defined by setting up the tree height as 0.80 times that of the maximum tree height, such that the tree canopy height (excluding the tree trunk height) was used for measuring the volume (square or circular pyramid) using Equations (1) and (2) for the polygon/bounding box and circle boundary area, respectively. The ratio of the canopy height with respect to the maximum tree height was finalized based on the manual measurements.

(1) $$V=\frac{1}{3}\left(A\times H\right)$$

(2) $$V=\pi r^{2}\times \frac{H}{3}$$

where *A* is the area of the polygon or bounding box, *r* is the radius of the circle, and *H* is the maximum height of the tree.

#### 2.4.2. Height and Volume Estimation from UAV-Based 3D Point Cloud

The .csv files converted from CloudCompare software (http://www.cloudcompare.org/ (accessed on 12 September 2021) were utilized for the tree height and canopy crown volume measurements. Using MATLAB software (R2016a, MathWorks Inc., Natick, MA, USA), a semi-automated algorithm was developed for processing the data to extract the architectural traits. The processing steps included the following: defining the matrix, rotating the point cloud using affine transformation to rectify the angular bias, followed by filtering unwanted 3D points. The individual trees were segmented by the boundary point, which was defined manually using the latitude (X) and longitude (Y) extent to segment the individual trees. Finally, the architectural traits of each tree from the 3D point cloud, including the height, were computed by subtracting the maximum Z value and minimum Z value (H = Z_max_ − Z_min_), and the volume (using voxel grid technique for the canopy crown only) was measured using a tetramesh function in MATLAB. The tetramesh function creates 3D tetrahedral shape across the boundary of the canopy crown by connecting the extent of the matrix. This measurement (canopy crown volume) was evaluated by comparing the extracted data with the manual measurements.

#### 2.4.3. Height and Volume Estimation from LiDAR System-Based 3D Point Cloud

The .pcap files were converted into .csv format using CloudCompare software. The data processing and extraction of tree height and canopy crown volume were performed using MATLAB (R2016a, MathWorks Inc., Natick, MA, USA). The background noise and ground points were eliminated prior to measuring the tree height and canopy crown volume. The acquired point cloud had an angle bias, which was corrected by affine transformation, and background noises were filtered by down sampling the point cloud. The individual trees were segmented by providing the width and length of the individual trees as the threshold. The tree height was measured by subtracting the minimum Z value from the maximum Z value (topmost level of the tree). The canopy crown volume was estimated using the voxel grid technique and the canopy crown alone was taken for estimation of the canopy crown volume instead of the whole tree. The voxel grid technique created the boundary that fit around the structure of the crown and measured the volume of the canopy crown.

### 2.5. Statistical Analysis

The Pearson’s correlation analysis was performed in R program (Version 4.1.1) to analyze the correlation coefficient at three levels of significance (5%, 1%, and 0.1%) between the ground reference data and the estimated tree height and canopy crown volume measurement under each scenario (individual and integrated nadir and oblique angles). As one tree in the second row was large, the data from this tree were eliminated so as to reduce its effect on the correlation results (*n* = 19). The data from each technique, including the ground reference data (manual measurements) were presented as a violin box plot to visualize the variation between the measurements for both the tree height and canopy crown volume. In the violin box plot, the median is represented as red dot, and the box ends represent the interquartile distance between the first and third quartile, and with the upper and lower values in the ends of the violin tips.

## 3. Results

### 3.1. UAV Data Analysis

#### 3.1.1. Tree Segmentation

**Figure 4:**
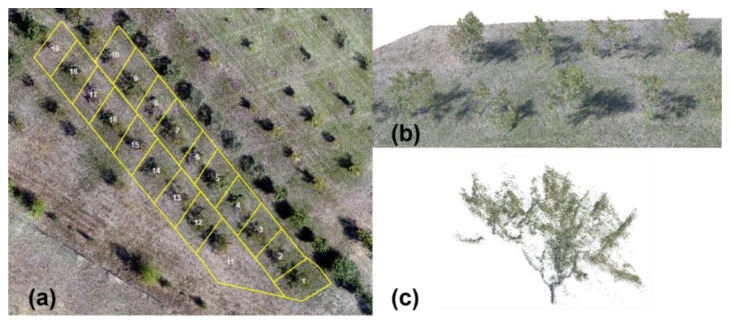
(**a**) Boundary representing the individual peach trees for segmentation, (**b**) 3D point cloud, and (**c**) 3D point cloud of an individual tree after segmentation.

A total of three UAV missions were conducted at three different sensor angle inclinations (90°, 45°, and 65°) to capture the stereo paired images. The obtained images were processed individually using images at each angle using photogrammetric software and were also processed by integrating the nadir (90°) and oblique angle (65° and 45°) images. The data types generated were DTM, DSM, orthomosaic, and 3D point cloud for each condition (45°, 65°, 90°, and integrated data). Using ArcMap 10.7, the orthomosaic imagery was used to identify the 19 peach trees and to segment the trees by creating the boundary for each tree as a shape file (.shp) for the 2D data analyses (Figure 4a). The 3D point cloud was segmented using the latitude–longitude extent of the individual trees (Figure 4b,c).

#### 3.1.2. Tree Height Estimation

**Figure 5:**
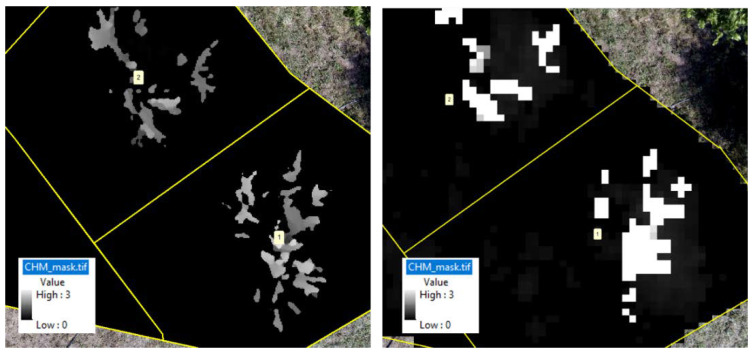
Canopy height model generated using the T1 (Pix4D) and T2 (point sampling) approaches.

The tree height was estimated from both the CHM and 3D point cloud data, generated for each of the sensor inclination angles and integrated datasets. Concerning the CHM approach, two data sources (T1 from Pix4D Mapper and T2 from the point sampling approach) were evaluated (Figure 5). A separate polygon of the individual tree boundary was created to capture canopy area, in addition to the bounding box and circle for the tree boundary using the canopy height model. The tree height was estimated using both CHMs (generated using T1 and T2 approaches) from the polygon, bounding box, and circular canopy area.

**Figure 6:**
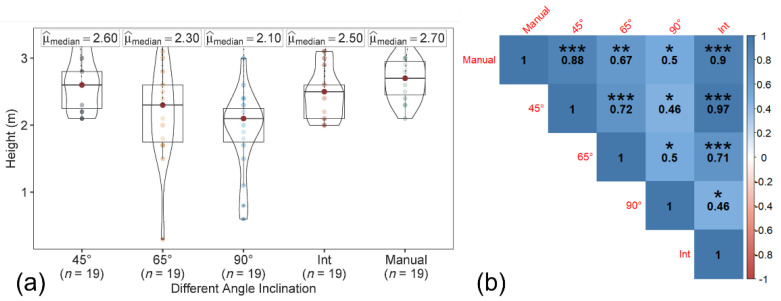
(**a**) Violin box plot showing the tree height range (m) and the (**b**) correlation matrix showing the correlation between the ground reference data and estimated T1-based tree height at individual angles (45°, 65°, and 90°) and the integration of nadir and oblique images. Significant probability level: * 0.05, ** 0.01, and *** 0.001.

**Figure 7:**
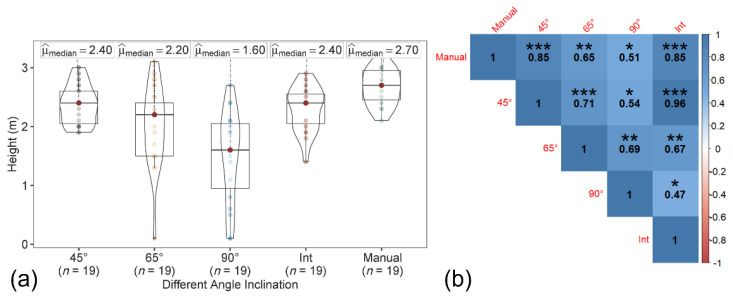
(**a**) Violin box plot showing the tree height range (m) and (**b**) correlation matrix showing the correlation between the ground reference data and estimated T2-based tree height at individual angles (45°, 65°, and 90°) and the integration of nadir and oblique images. Significant probability level: * 0.05, ** 0.01, and *** 0.001.

The maximum tree height based on the polygon, bounding box, and circular canopy area did not change for both the T1 and T2 datasets. The manually measured peach tree height (ground reference data) ranged from 2.13 to 3.05 m, with a median of 2.70 m. In both T1-based and T2-based tree height estimations (Figure 6 and Figure 7), among the datasets, the tree height from image acquired at 45° and the integrated nadir and oblique images showed the similar trend and high correlation with the ground reference data. The correlation coefficient of the T1-based tree height estimation with ground reference data was slightly higher than the T2-based tree height.

**Figure 8:**
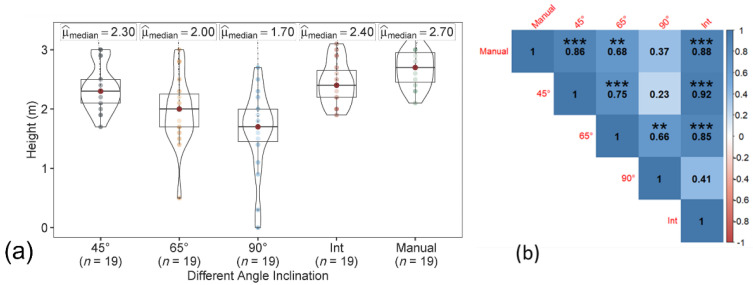
(**a**) Violin box plot showing the tree height range (m) and (**b**) correlation matrix showing the correlation between the ground reference data and UAV point cloud-based estimated tree height at individual angles (45°, 65°, and 90°) and the integration of nadir and oblique images. Significant probability level: ** 0.01, and *** 0.001.

In the 3D point cloud dataset, similar to the T1- and T2-based approaches, the tree height from the data acquired at the angle of 45° and the integrated nadir and oblique images were closely associated with the ground reference data, than with the data acquired at 65° and 90° (Figure 8). The tree height from the integrated datasets was also highly correlated to the 45° datasets (*r* ≥ 0.92) in all three approaches (T1, T2, and points cloud).

**Figure 9:**
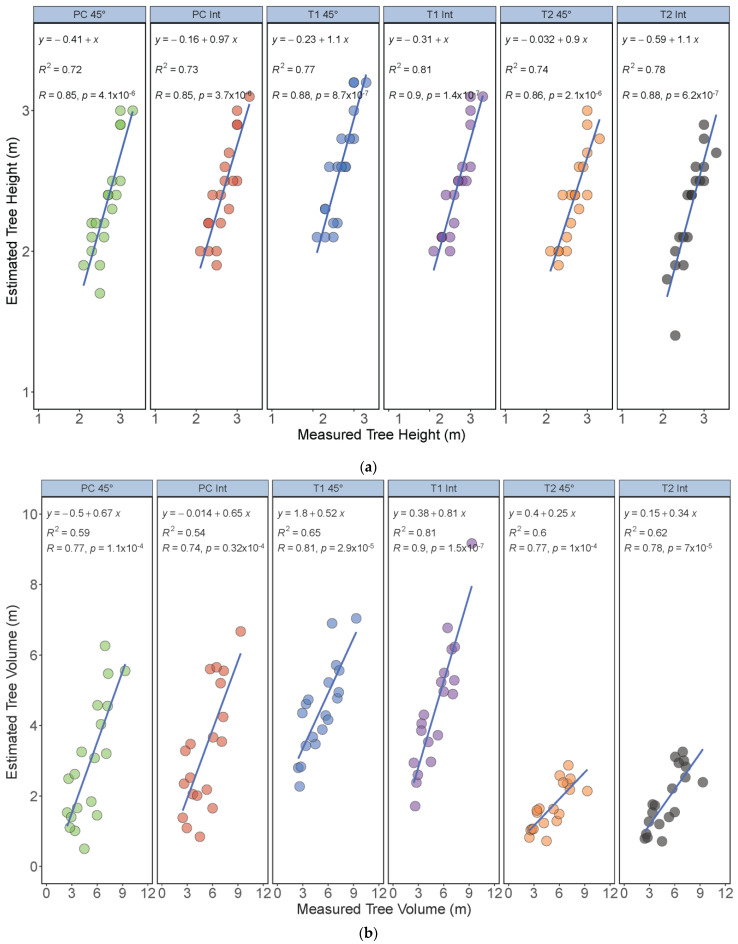
Relationship between the ground reference data and estimated tree height (**a**) and volume (**b**) acquired at 45° and the integrated nadir and oblique datasets using T1 (circular), T2 (polygon), and point cloud datasets, respectively.

Among all of the three techniques (T1, T2, and point cloud), the data extracted from the 45° and integrated nadir and oblique datasets showed a higher correlation and significant *p*-value. Comparing the T1, T2, and point cloud-based approaches, the correlation with ground reference data was marginally higher in the T1-based approach (45° and integrated datasets), while the median tree height was closest to the ground reference data in the 45° dataset. The slope in most cases (T1, T2, and point cloud, 45° and integrated datasets) was close to one (Figure 9). Given the efforts in data acquisition, processing, and analysis, a 45° sensor inclination angle may be beneficial. Overall, for orchard peach trees, a higher accuracy tree height can be estimated at a 45° sensor inclination angle.

#### 3.1.3. Canopy Crown Volume Estimation

**Figure 10:**
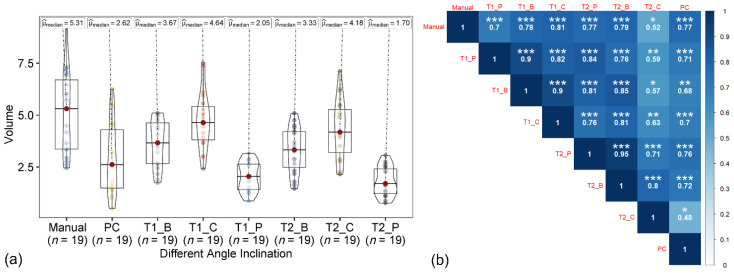
(**a**) Violin box plot showing the canopy crown volume range (m) and the (**b**) correlation matrix showing correlation between ground reference data and estimated volume with a 45° sensor angle dataset using T1, T2, and point cloud (PC) UAV-based approaches with canopy area estimated using polygon, box, and circular canopy area. Significant probability level: * 0.05, ** 0.01, and *** 0.001.

**Figure 11:**
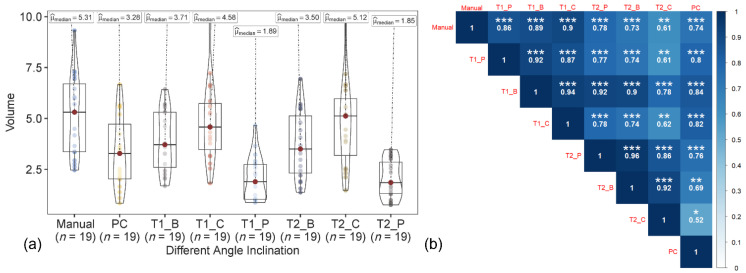
(**a**) Violin box plot showing the canopy crown volume range (m) and the (**b**) correlation matrix showing the correlation between ground the reference data and estimated volume with the integrated sensor angle dataset using the T1, T2, and point cloud (PC) UAV-based approaches with canopy area estimated using the polygon, box and circular canopy area. Significant probability level: * 0.05, ** 0.01, and *** 0.001.

In the T1- and T2-based approaches, the volume of the canopy crown was measured using the canopy height (excluding trunk length) instead of the whole tree height for the polygon, bounding box, and circular canopy area (Figure 10 and Figure 11). Only 45° and the integrated datasets were analyzed, as these datasets resulted in the most accurate results for the tree height estimation. Among the volume extracted from the polygon, bounding box, and circle-shaped canopy area from 45° and the integrated datasets, using the circular and bounding box area with the integrated dataset showed a higher similarity to the ground reference data than the polygon-shaped canopy areas for the T1-based approach. However, the polygon showed similar relationship to the ground reference data with both datasets (45° and integrated) for the T2-based approach. The best results (high correlation with ground reference data) were from the volume of the canopy crown extracted from the integrated nadir and oblique images dataset (especially with circular canopy area) with the T1-based approach.

**Figure 12:**
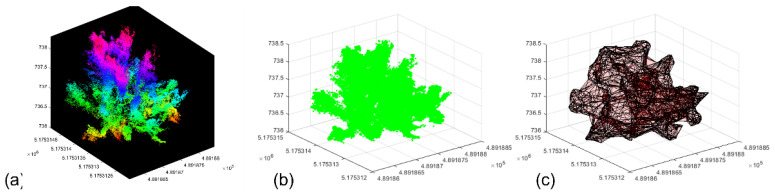
(**a**,**b**) Tree canopy crown delineation and (**c**) voxel grid model creating the boundary to measure the volume of the canopy crown.

In the 3D point cloud dataset, the volume was extracted by delineating the canopy crown from the tree height by providing the threshold for each tree and measuring the volume of the boundary as the voxel grid (Figure 12). The 3D point cloud-based canopy crown volume showed the highest correlation to the ground reference data with the 45° dataset, followed by the integrated nadir and oblique images dataset. The lower correlation for the T1- and T2-based approaches could be associated with the un-matching ground reference data [26]. Among the three techniques (T1, T2, and 3D point cloud), the T1-based approach with integrated nadir and oblique datasets showed the strongest relationship with ground reference data.

### 3.2. LiDAR Data Analysis

**Figure 13:**
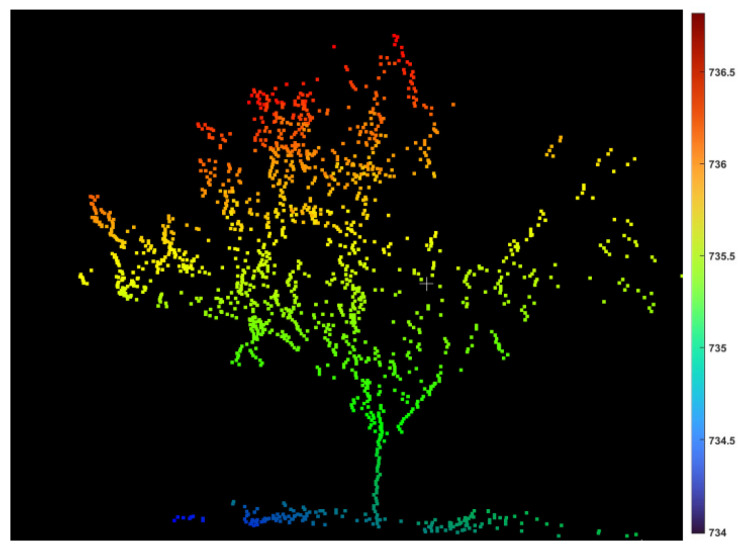
Visualization of a representative peach tree 3D data captured using the 3D LiDAR system. The color scale refers to height (z) data in m above sea level.

**Figure 14:**
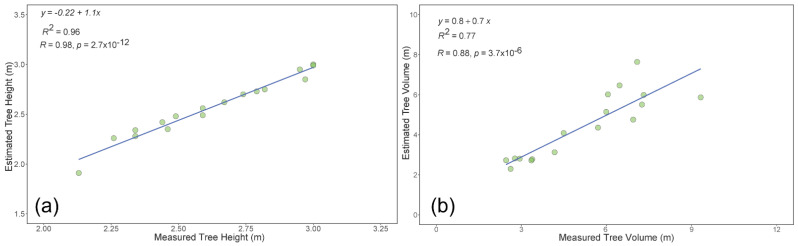
Relationship between the ground reference data with the estimated tree height (**a**) and tree volume (**b**) data acquired using the point cloud dataset from the LiDAR system.

In addition to the UAV data, the LiDAR data from 17 trees were acquired to measure the tree height and canopy crown volume (Figure 13). The canopy crown volume was estimated using the voxel grid technique. The estimated tree height (*r* = 0.98/R^2^ = 0.96) and canopy crown volume (*r* = 0.87 and R^2^ = 0.77) showed a high and strong correlation with the ground reference data (Figure 14).

The comparison of the estimated tree height from UAV data for 17 trees with LiDAR data indicated that with all datasets, the correlations coefficients were similar to those with the ground reference data. This could be because of the strong correlation between the ground reference and the LiDAR-based tree height data. However, similar results were not observed for the canopy crown volume. The canopy crown volume (from integrated and 45° datasets) extracted from the UAV data were not as highly correlated with the LiDAR data compared with the ground reference data. This could be the result from the nature of the ground reference data measurements, where the UAV data could be more directly related to the ground reference data than the LiDAR data. Further evaluation on these aspects needs to be performed.

## 4. Discussions

In recent years, researchers have utilized sensor-based technology coupled with UAV systems in various applications for monitoring and decision-making in orchard management [29]. UAV sensing techniques have been used to extract the architectural traits in tree crops, such as canopy height, volume, and other structures [30,31]. This study focused on the importance and need for optimizing flight and sensor parameters in the extraction of architectural traits such as tree height and canopy crown volume. In general, UAV flight variables such as flight altitude, image overlap, flying direction, flying speed, and solar elevation and azimuth affect the image quality [32].

In this study, three UAV missions at different sensor inclination angles (45°, 65°, and 90°) and the integration of all the angles (nadir and oblique) to extract the accurate measurement of the architectural traits of the individual trees was assessed using three different techniques. The results showed that the features extracted from the images collected at a sensor inclination of 45° and the integration of nadir and oblique images exhibited a similar correlation with the ground reference data and measurement results, followed by angles of 65° and 90°. Similar research findings showed that the images acquired at oblique angles improved the 3D reconstruction of the forest canopy. The results showed that the oblique images increased the understory point density and accuracy of the canopy crown percentage and the tree height increased by 33% and 50%, respectively [33].

In the tree height measurements, all UAV-based approaches (T1, T2, and point cloud) showed similar results with 45° and the integrated datasets. The best accuracy was found for those derived from LiDAR data, although the results from the UAV-based approaches were decent with a slope close to one. Regarding the canopy crown volume, Pix4D derived DTM showed a strong correlation with the ground reference data with the integrated nadir and oblique datasets. Overall, the approach of collecting oblique images and integrating datasets for measuring architectural traits can improve precision. The most significant improvements were found when including the oblique imagery for canopy representation [34]. The 3D LiDAR point cloud also showed a strong relationship with the ground reference data.

## 5. Conclusions

High-resolution RGB imagery acquired at different nadir and obliques images were processed and orthomosaic, DSM, DTM, 3D UAV point cloud, and LiDAR point cloud datasets were generated to measure the peach tree height and canopy crown volume. The study evaluated the tree height and canopy crown volume estimation accuracy at each angle and the integrated nadir and oblique imagery datasets. The results were validated, with statistical analysis performed to compare the extracted features to the ground reference data (manual measurements). Overall, the images acquired at 45° and the integrated nadir and oblique images yielded accurate tree height estimations using all three UAV-based approaches (T1, T2, and point cloud), with the integrated dataset proving useful in extracting the accurate canopy crown volume. The 3D LiDAR point cloud also showed a very high correlation in terms of tree height and canopy crown volume. The change in sensor inclination angles and the integration of multiple angular datasets did affect the measurement accuracy. Nevertheless, further studies are recommended to assess the change in flying altitude with respect to different sensor inclination angles to study the effect on the accuracy of the estimation of tree canopy attributes.

## Figures and Tables

**Table 1:** Summary of parameters used for UAV flight.

Mission	Type	Altitude	Sensor Inclination	GSD (cm/Pixel)	Flight Speed	Overlap (Forward/Side)
1	Double grid	15 m	90°	0.29	2.5 m/s	80%
2			65°	0.37		
3			45°	0.81		

## Data Availability

The data presented in this study are available upon request from the corresponding author.
